# Factors Related to Severity, Hospitalization, and Mortality of COVID-19 Infection among Patients with Autoimmune Diseases

**DOI:** 10.3390/tropicalmed8040227

**Published:** 2023-04-18

**Authors:** Alvina Widhani, Sukamto Koesnoe, Suzy Maria, Annisa Layalia Widjanarko, Teguh Harjono Karjadi, Anshari Saifuddin Hasibuan, Evy Yunihastuti, Iris Rengganis, Samsuridjal Djauzi

**Affiliations:** 1 Allergy and Clinical Immunology Division, Department of Internal Medicine, Faculty of Medicine, Universitas Indonesia/Cipto Mangunkusumo Hospital, Jakarta 10430, Indonesia; sukamto_koesnoe@yahoo.com (S.K.); suzyduri@gmail.com (S.M.); tghsemar59@gmail.com (T.H.K.); sorihsb@gmail.com (A.S.H.); evy.yunihastuti@gmail.com (E.Y.); irisrengganis@yahoo.com (I.R.); samsuridjal@yahoo.com (S.D.); 2 Department of Internal Medicine, Universitas Indonesia Hospital, Depok 16424, Indonesia

**Keywords:** COVID-19, autoimmune disease, severity, hospitalization, mortality

## Abstract

Patients with an autoimmune disease could be at higher risk of a poor outcome when contracting COVID-19 infection due to aberrant immune responses and use of immunosuppressant therapies for chronic autoimmune treatment. Here, we conducted a retrospective study to identify the factors related to severity, hospitalization, and mortality among patients with autoimmune diseases. We found 165 cases of patients with pre-existing autoimmune diseases who had contracted COVID-19 between March 2020 and September 2022. Data on demographical characteristics; autoimmune diagnosis and treatment; COVID-19 vaccination status; and time, severity, and outcome of COVID-19 infection were collected. Most of the subjects were female (93.3%) and autoimmune diagnoses included systemic lupus erythematosus (54.5%), Sjogren’s syndrome (33.5%), antiphospholipid syndrome (23%), vasculitis (5.5%), autoimmune thyroid disease (3.6%), rheumatoid arthritis (3.03%), and inflammatory bowel disease (3.03%) among other autoimmune diseases. There were four COVID-19-related deaths in this study. Factors associated with moderate to severe COVID-19 infection in patients with autoimmune diseases included not being vaccinated against COVID-19, taking a steroid of ≥10 mg prednisone-equivalent per day, and having a cardiovascular disease. Taking a steroid of ≥10 mg prednisone-equivalent per day was also associated with hospitalization in the event of COVID-19 infection, while cardiovascular diseases also showed a significant correlation to mortality in patients with autoimmune diseases who had been hospitalized with COVID-19 infection.

## 1. Introduction

Coronavirus disease 2019 (COVID-19), a respiratory infection caused by severe acute respiratory syndrome coronavirus 2 (SARS-CoV-2), is a global pandemic that was first reported in Wuhan, Hubei, China in December 2019. SARS-CoV-2 has been reported to have a higher affinity to angiotensin-converting enzyme 2 (ACE-2) receptors than SARS-CoV, and thus is more powerful and more easily transmitted than SARS-CoV and MERS-CoV [1]. The virus rapidly spread around the world and has become a global pandemic. By 25 January 2023, the World Health Organization (WHO) had reported 664,873,023 confirmed COVID-19 cases with 6,724,248 deaths globally [2]. As of 22 November 2022, Indonesia’s Ministry of Health reported a total of 6,612,673 confirmed COVID-19 cases, with 159,422 deaths and 6,393,664 recovered cases at the national level [3]. Studies have reported various degrees of severity, ranging from asymptomatic infection to severe pneumonia with respiratory failure, with the mortality rate increasing up to 61.5% in severe and critical cases [1,4]. Patients of older ages (≥65 years) and those with comorbidities, such as cardiovascular disease, acute kidney injury, chronic kidney disease, and diabetes mellitus, have a higher risk of mortality [4,5,6]. Studies have found increased inflammatory biomarkers, including those of C-reactive protein (CRP), erythrocyte sedimentation rate (ESR), and procalcitonin (PCT) in COVID-19 death cases. These biomarkers confirm the cytokine storm and immune damage resulting in multiple organ dysfunction due to SARS-CoV-2 infection [5,6]. Multiple organ dysfunction increases the risk of morbidity and mortality in COVID-19 patients [6].

Clinical and radiologic findings of COVID-19 resemble findings characterizing the pneumonia of autoimmune and autoinflammatory diseases, which have shown that SARS-CoV-2 can drive the activation of aberrant innate and acquired immune responses [7]. A study in China reported prevalence of 20% anti-52 kDa SSA/Ro antibody, 25% anti-60 kDa SSA/Ro antibody, and 50% antinuclear antibody in COVID-19 patients, suggesting the existence of autoimmune phenomena in COVID-19 subjects [8]. These findings raised concerns about patients affected by autoimmune diseases during the COVID-19 pandemic.

A 2020 study in Italy discovered a similar pattern of risk of COVID-19 between autoimmune patients and the general population [9]. However, a multicenter study in China indicated a possible higher risk of SARS-CoV-2 infection in patients with rheumatic disease taking DMARDs (disease-modifying antirheumatic drugs) [10]. Similarly, a study in the United Kingdom also found a higher risk of COVID-19-related death in patients with rheumatic diseases. In addition to older age and the presence of comorbidities, autoimmune disease activity has been shown to have a significant association with COVID-19-related death [11]. Discontinuation of immunosuppressive medications was discovered among patients with autoimmune diseases, mainly due to fear of the immunosuppressive effects of their medications [9,12]. However, patients with autoimmune diseases taking hydroxychloroquine have been shown to have a lower risk of COVID-19 infection compared to those taking other immunosuppressants [10].

To our knowledge, there has not been any information reported regarding clinical characteristics and outcomes of COVID-19 infection in autoimmune patients in Indonesia. Our study, therefore, aims to identify outcomes and factors associated with severity, hospitalization, and mortality of COVID-19 in patients with autoimmune diseases.

## 2. Materials and Methods

This observational study was conducted at Cipto Mangunkusumo General Hospital and Universitas Indonesia Hospital, Indonesia. Data were obtained from October 2021 until September 2022. Patients with autoimmune diseases who visited the allergy and clinical immunology outpatient clinics were screened for previous COVID-19 history. We included patients with autoimmune diseases who had a previous history of COVID-19 confirmed by positive SARS-CoV-2 PCR or rapid antigen results. Patients who refused informed consent were excluded. After giving consent, the participants were given a questionnaire from which demographical data and COVID-19 clinical information were obtained. Clinical data were also obtained from electronic medical records. We also collected retrospective data from medical records of our autoimmune patients who had been hospitalized because of COVID-19. Patients that had been infected by COVID-19 more than once were counted as multiple individual COVID-19 cases.

Information on demographical data, diagnosis, and treatment of autoimmune diseases and comorbidities, COVID-19 vaccination status, and history of COVID-19 including severity, hospitalization, and outcomes (deceased or alive) was collected. COVID-19 severity was classified according to Indonesia’s Ministry of Health’s Guidelines for COVID-19 Management: mild (patients with fever, cough, myalgia, anosmia, ageusia, and oxygen saturation of >95%); moderate (patients with fever, cough, shortness of breath, and oxygen saturation of 93–95%); and severe (patients with signs of pneumonia with at least one of the following: respiratory rate more than 30 bpm and/or oxygen saturation of <93%). All patients’ data were kept confidential.

Ethical clearance was obtained from the Medical Research Ethical Committee, Faculty of Medicine, Universitas Indonesia (KET-1018/UN2.F1/ETIK/PPM.00.02/2021) and Universitas Indonesia Hospital (S-074/KETLIT/RSUI/XII/2021).

Collected data were analyzed using IBM SPSS Statistics ver. 29.0.0.0 2022. Normality of data was analyzed using the Kolmogorov–Smirnov test. Numerical data were presented as means and standard deviations if normally distributed or medians (minimum, maximum value) if abnormally distributed. Categorical data were presented as subjects (n) and proportions. Bivariate analyses were determined using Chi-square and Fisher’s exact tests as appropriate and presented as odds ratios with a 95% confidence interval and as *p* values where appropriate. *p* values of <0.05 were considered statistically significant. Multivariate logistic regression was conducted to determine the factors associated with COVID-19 outcome in autoimmune patients. Independent variables with *p* values of <0.250 were included in the multivariate analysis.

## 3. Results

### 3.1. Characteristics of Subjects

From March 2020 until September 2022, there were 165 cases of patients with autoimmune diseases who had also contracted COVID-19. The characteristics of subjects with a history of COVID-19 infection are presented in Table 1. Median age was 34 (18; 72) years old and 93.3% of the subjects were female. Most of the subjects had a normal body mass index (BMI) with the median BMI being 22.9 (12.3; 42.3). The most common comorbidities in the subjects of our study were hypertension and chronic kidney disease (18.3% and 11%, respectively). The percentages of subjects with an autoimmune diagnosis were 54.5%, 33.5%, 23%, 5.5%, and 3.6% for SLE, Sjogren’s syndrome, antiphospholipid syndrome, autoimmune vasculitis, and autoimmune thyroid disease, respectively. There were 60 (36.4%) subjects that had multiple autoimmune diagnoses.

Thirty-eight (23%) subjects only received corticosteroids or a steroid sparing agent as their autoimmune treatment; 108 (65.5%) subjects were on combination therapy, taking either a steroid with a steroid sparing agent or a steroid with more than one steroid sparing agent; and 19 (11.5%) were not taking any immunosuppressants. About 71.5% of subjects had been treated with a corticosteroid for an autoimmune disease, with the median dose of corticosteroid being 5 mg prednisone-equivalent per day (0.63; 78.13 mg/day). Most of them (61.0%) were taking corticosteroids at a dose less than 10 mg prednisone-equivalent per day. Besides corticosteroids, most of our subjects were given mycophenolate mofetil/mycophenolic acid (MMF/MPA) or hydroxychloroquine (41.2% and 41.2%, respectively). Other routine medications included antiplatelet drugs (13.3%) and vitamin D3 supplements (27.3%) with a median dose of 2000 IU/day (1000; 5000 IU/day).

About 42.4% of our subjects had received a COVID-19 vaccine, with the majority (40%) receiving CoronaVac. Most of the subjects had received their COVID-19 vaccination after having been infected with COVID-19 (48.5%).

Most of the patients (79.4%) in our study experienced mild COVID-19 infection. For most of the patients who were receiving a corticosteroid for their autoimmune treatment (106 subjects, 89.8%), medication was continued at the same dose during the SARS-CoV-2 infection. Five patients had their steroid dose increased, two patients had the dose decreased, and the other two subjects had it stopped. Among the patients treated with MMF/MPA for autoimmune disease, about 30 of them (44.1%) had it stopped during COVID-19 infection and two patients were instructed to decrease their MMF/MPA dose, while about 36 patients (52.9%) continued with the same dose of MMF/MPA. Most of the subjects (29 of 36 patients) that continued MMF/MPA treatment with the same dose during COVID-19 infection experienced only mild COVID-19 infection. Among the 11 patients receiving azathioprine, 7 patients (63.6%) had treatment stopped, while the others continued taking azathioprine. All patients on azathioprine had mild COVID-19 infections. Most patients (62 subjects, 91.2%) who were receiving hydroxychloroquine for their autoimmune disease continued taking it with the same dose during COVID-19 infection. However, one patient had the dosage increased, one had it decreased, and four patients (5.9%) had treatment stopped. Of the patients who were receiving methotrexate or tacrolimus, one subject had medication stopped, while two others continued with medication. All patients who continued methotrexate or tacrolimus treatment had mild COVID-19 infections. All patients taking cyclophosphamide had treatment stopped when they contracted COVID-19.

**Figure 1:**
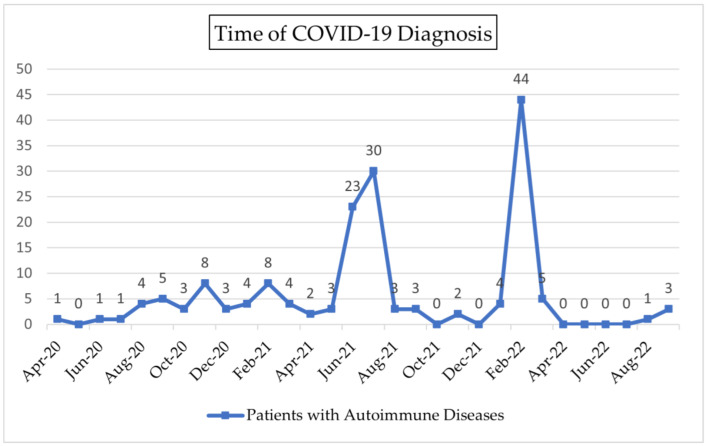
Time of COVID-19 Diagnosis.

Most of our subjects (37%) were diagnosed with COVID-19 infection (Figure 1) during the Delta period (from June 2021 until December 2021). About 34.5% of the patients were diagnosed with COVID-19 infection during the Omicron period (from December 2021 onward). Predominant COVID-19 variants were based on sequencing data reported by Indonesia’s Ministry of Health [13,14,15]. Most of our subjects (63%) self-isolated during COVID-19 infection. Only four subjects in our study died.

### 3.2. Factors Associated with Severity, Hospitalization, and Mortality of COVID-19 in Patients with Autoimmune Diseases

Bivariate analysis was used to identify factors associated with moderate–severe COVID-19 infection, hospitalization, and mortality among patients with autoimmune diseases who contracted COVID-19. Factors significantly associated with moderate–severe COVID-19 infection (Table 2) were not being vaccinated against COVID-19 (*p* = 0.021), having diabetes mellitus (*p* = 0.019), and taking a corticosteroid of ≥10 mg prednisone-equivalent per day (*p* = 0.010). Multivariate analysis revealed that patients who had not been vaccinated against COVID-19 were taking a corticosteroid of ≥10 mg prednisone-equivalent per day, or had any cardiovascular disease had higher odds of developing moderate to severe disease (OR 5.65 (1.32–24.16), *p* = 0.019; OR 3.01 (1.06–8.48), *p* = 0.037; and OR 7.22 (1.34–39.00), *p* = 0.022, respectively).

Most of our subjects (63%) self-isolated when they contracted COVID-19. Bivariate analysis indicated that the factors significantly associated with hospitalization as a result of COVID-19 infection were not being vaccinated against COVID-19 (*p* = 0.025), taking a corticosteroid of ≥10 mg prednisone-equivalent per day (*p* < 0.001), and taking tacrolimus as routine immunosuppressant therapy (*p* = 0.049). Factors significantly associated with self-isolation during COVID-19 therapy were taking hydroxychloroquine as chronic autoimmune treatment and taking a vitamin D3 supplement (*p* = 0.019 and *p* = 0.041, respectively). Multivariate analysis revealed that patients with autoimmune diseases that were taking a corticosteroid of ≥10 mg prednisone-equivalent per day had higher odds of hospitalization resulting from COVID-19 infection (OR 5.36 (2.10–13.65), *p* < 0.001).

There were 4 mortalities out of the 165 COVID-19 cases in our study (Table 3). All were SLE patients, needed hospitalization, and had not been vaccinated against COVID-19. Bivariate analysis (Table 2) showed that the factor significantly associated with mortality was having cardiovascular disease (OR 11.38 (1.48–87.58), *p* = 0.042).

## 4. Discussion

As COVID-19 became a global pandemic, its rapid transmission became a primary health concern in Indonesia. As the virus undergoes genetic mutations, the emergence of variants of concern could contribute to the severity of disease. Patients with autoimmune diseases are at risk of developing severe COVID-19 due to the nature of the disease and the use of immunosuppressant therapies [10]. In the event of infection, patients with autoimmune diseases have dysregulated innate and acquired immune responses that lead to overproduction and overresponse, mediated by pro-inflammatory cytokines such as TNF-α, IL-6, IL-Iβ, IL-17, and IL-18 [7]. This study analyzed factors contributing to the severity, hospitalization, and outcome of COVID-19 among patients with autoimmune diseases in two hospitals in Jakarta, an area that contributed greatly to surges in cases during the period of two variants of concerns in Indonesia, Delta and Omicron. Our subjects were most frequently infected with the Omicron variant in February 2022, which followed the Delta variant in June–July 2021.

Vaccines for COVID-19 started development in 2020 [16]. The COVID-19 vaccination program in Indonesia was conducted in four phases: (I) first phase (January–April 2021) for health care workers, health-care support personnel, and medical students who had begun actively practicing in health-care facilities; (II) second phase (January–April 2021) for public service officers, including the army and police officers, and the elderly (≥60 years); (III) third phase (April 2021–March 2022) for the socially, economically, and geospatially susceptible general public due to a high risk of infection; and (IV) fourth phase (April 2021–March 2022) for the general public, which was when vaccine was more widely available. In March 2021, the Indonesian Society of Internal Medicine recommended COVID-19 vaccination for patients with autoimmune diseases in stable conditions if they could show a letter of recommendation from their physician [17].

Our study revealed that patients who had not been vaccinated against COVID-19 had higher odds of developing moderate to severe disease (OR 5.65 (1.32–24.16), *p* = 0.019) and higher odds of hospitalization due to COVID-19 (OR 2.63 (0.99–6.95), *p* = 0.050). All four of our death cases died during COVID-19 hospitalization and all four were not vaccinated against COVID-19. A 2022 meta-analysis of 51 studies found a 97.2% vaccine effectiveness for the prevention of COVID-19-related hospitalization, 97.4% effectiveness for the prevention of severe COVID-19, and 99.0% effectiveness for the prevention of COVID-19-related death in fully vaccinated people. In the general population, 86.1% vaccine effectiveness for the prevention of COVID-19 infection has been reported [18]. Regarding the Omicron variant, some studies found that vaccine effectiveness was reduced for symptomatic infection, but a booster dose of overall vaccines (mRNA, BNT162b2, mRNA-1273, or ChAdOx1 vaccines) could provide better protection against severe Omicron variant COVID-19 infection [19,20]. A 2022 study in Spain found that patients with an autoimmune disease have reduced humoral immune response to primary mRNA vaccines (seroconversion rate of 80%) compared to healthy controls (100%) [21].

It has also been reported that patients with autoimmune rheumatic diseases are no more likely to develop adverse events after COVID-19 vaccination with the ChAdOx1 vaccine than the general population are [22]. Similarly, patients with chronic inflammatory diseases were found to experience mild systemic side effects after mRNA vaccines, with no disease flares or immunosuppressant dose adjustments [23]. Patients with systemic autoimmune myopathies that received anti-SARS-CoV-2 inactivated vaccine also showed moderate IgG seroconversion rate, but only experienced mild side effects following vaccination with similar frequencies compared to healthy control group [24]. Considering there is a benefit to reducing the severity of COVID-19 infection, patients with autoimmune diseases have been encouraged to get vaccinated during remission or low disease activities [25].

Our study found that patients with an autoimmune disease taking a prednisone-equivalent corticosteroid of ≥10 mg/day showed higher odds of severe COVID-19 infection and COVID-19-related hospitalization. Our study supports a 2020 study that found increased likelihood of COVID-19 hospitalization in patients with rheumatic diseases taking ≥10 mg/day of prednisone-equivalent glucocorticoids that are registered on the COVID-19 Global Rheumatology Alliance Registry [26]. Another 2021 study of 12 matched cohorts in South Korea found high doses of a corticosteroid (≥10 mg/day) to be an independent risk factor for the development of severe COVID-19 and COVID-19-related death, which is similar to the current study [27]. Similar to our findings, both studies found no association between the use of immunosuppressants and severe COVID-19 infection, COVID-19-related hospitalization and death in patients with rheumatic disease. This is in contrast to a 2020 study in Spain that found that autoimmune inflammatory rheumatic disease treatment with a steroid does not increase risk for COVID-19 hospital admission [28]. Considering that corticosteroids are still the main treatment for patients with autoimmune diseases, it is important to taper corticosteroid dose to the minimal optimal dose that can still control disease activity. Early use of a steroid sparing agent, according to indication, can help to achieve this target. Hydroxychloroquine is one option of a steroid sparing agent which has a lower infection risk than other immunosuppressive agents [29]. In our study, chronic use of hydroxychloroquine for autoimmune treatment was associated with a lower likelihood of COVID-19-related hospitalization in bivariate analysis, although the multivariate analysis did not show a significant difference. This is in accordance with previous studies which have shown that use of hydroxychloroquine in patients with autoimmune diseases has no significant association with COVID-19 hospitalization [26]. Hydroxychloroquine has been used in autoimmune diseases due to its immunomodulatory properties, and during the pandemic it was repurposed due to its potential to help reduce inflammation and prevent the catastrophic cytokine storm [29,30]. However, a more recent controlled trial found no difference in seroconversion rates of SARS-CoV-2 by 28 days, and another found no efficacy in the use of hydroxychloroquine for the post-exposure prophylaxis of COVID-19 [29,31].

From bivariate analysis it can be seen that COVID-19 infection-related hospitalization in our study was significantly lower among patients taking routine vitamin D3 supplementation compared to patients who were not, even though the difference was not significant in multivariate analysis. Patients taking routine vitamin D3 supplementation also had a lower likelihood of developing moderate–severe COVID-19 infection compared to patients who were not taking vitamin D3 supplementation, although this difference was not significant. A 2021 metanalysis conducted in Italy found that low 25-hydroxyvitamin D (25 (OH)D3) levels (≤30 ng/mL) at the time of COVID-19 hospitalization were associated with more severe COVID-19 infection during hospitalization [32]. Another 2021 meta-analysis on 39 studies also revealed that vitamin D deficiency was associated with increased risk of severe SARS-CoV-2 infection and COVID-19 hospitalization [33]. This could be attributed to the modulatory effect of vitamin D on excessive Th1 activity and production of proinflammatory cytokines during cytokine storm, and also on the neutrophil activity that orchestrates inflammation of lung alveoli [32,33]. Vitamin D, in its active form 1,25 (OH)2D3, could also directly reduce proliferation of B cell and immunoglobulin production [34]. Low 25 (OH)D3 levels in systemic autoimmune diseases, such as SLE and rheumatoid arthritis, have been associated with worse disease activities [35]. Vitamin D3 supplementation could be beneficial in these diseases, and this might explain the protective effect of vitamin D3 supplementation in COVID-19 severity and hospital admission.

We had four cases of COVID-19-related death (2.42%). Our study shows that, besides the higher risk of moderate-severe COVID-19 infection, the presence of any cardiovascular disease, such as coronary artery disease or heart failure, could increase the risk of COVID-19 mortality among patients with autoimmune diseases. A systematic review and meta-analysis showed that cardiovascular disease was one of the factors related to severe COVID-19 infection [36]. Our result was also in accordance with a 2020 study on the general population that found a higher risk of in-hospital death in patients with coronary heart disease. Pre-existing cardiovascular diseases, such as ischemic heart disease, could increase the risk of cardiac events after pneumonia related to COVID-19 [4]. A meta-analysis showed that preexisting cardiovascular disease is an independent risk factor associated with adverse events among COVID-19 patients [37]. Previous studies have found increased cardiac markers in COVID-19 death cases [4,5]. As SARS-CoV-2 binds to ACE2 receptors in the heart, lungs, and kidneys, it contributes to the development of multi-organ dysfunction in the event of pro-inflammatory cytokine response [4,5,38]. Our results contrast with some studies that found no significant association between COVID-19-related mortality and cardiovascular diseases among the general population [39,40].

In our study, there were nine autoimmune patients with diabetes mellitus and five of them suffered moderate-severe COVID-19 according to the classification used by the National Ministry of Health in Indonesia. Bivariate analysis in our study revealed that autoimmune patients with diabetes mellitus have a significant risk of developing severe COVID-19 (OR 5.47 (1.38–21.65), *p* = 0.019), although multivariate analysis showed no significant difference. Similarly, a meta-analysis of 33 studies showed that diabetes patients have a higher risk of developing severe COVID-19 infection than the general population (OR 2.75 (95% CI 2.09–3.62), *p* < 0.01) [41]. Another 2020 study of 123 autoimmune patients also revealed that the presence of comorbidities, such as diabetes mellitus, could increase the risk of hospital admission related to COVID-19 [28]. Phagocytic cell dysfunction, inhibition of neutrophil chemotaxis, and impaired T-cell mediated immune response in patients with diabetes could result in catastrophic systemic inflammation. Disturbance of glucose homeostasis, inflammation and activation of the renin-angiotensin-aldosterone system in diabetes mellitus could contribute to severe forms of COVID-19 infection [39].

Thrombosis and coagulopathy are complications that can occur in severe COVID-19 infection. The incidence of thromboembolic events, such as venous thromboembolism, in patients with COVID-19-related coagulopathy is 31% [42]. Similarly, antiphospholipid antibodies (aPL) were found to be present in severe COVID-19 infection, but they appeared to be only transient [43]. Pneumocytes that produce surfactants are primary binding sites for SARS-CoV-2 because they contain large amounts of ACE2 receptors. Necrosis of pneumocytes following SARS-CoV-2 infection exposes pulmonary surfactants, which are rich in phospholipid-binding proteins, to the immune system [44,45]. In our study, patients with antiphospholipid syndrome showed a greater likelihood of severe–moderate COVID-19 infection, hospitalization, and mortality compared to patients without antiphospholipid syndrome, although the differences were not statistically significant.

There are several limitations to our study: Firstly, the small sample size and the lack of recruitment of subjects from other provinces of Indonesia. Our study also has sampling bias due to consecutive sampling. Our subjects comprise patients with autoimmune diseases who routinely visit the outpatient clinics or were hospitalized in our hospitals. Our patients who were hospitalized in other hospitals may have died from COVID-19 infection and this may be unrecorded. Another limitation is that we could not measure disease activity due to heterogenous autoimmune diagnosis. This could be a confounding factor potentially affecting vaccination status, steroid dose, and severity of COVID-19 infection. Patients with autoimmune diseases that have high disease activity might avoid COVID-19 vaccination, have higher doses of corticosteroids, and experience more severe COVID-19 infections.

## 5. Conclusions

Not being vaccinated against COVID-19, taking a steroid of ≥10 mg prednisone-equivalent per day, and having any cardiovascular disease were found to be associated with moderate to severe COVID-19 infection in patients with autoimmune diseases. Taking a steroid of ≥ 10 mg prednisone-equivalent per day was also found to be associated with hospitalization due to COVID-19 infection, while cardiovascular disease was found to be significantly related to mortality in patients with autoimmune diseases who had been hospitalized with COVID-19 infection.

## Figures and Tables

**Table 1:** Baseline Characteristics of Subjects (*n* = 165).

Variables	*n* (%)
Sex	
Female	154 (93.3)
Male	11 (6.7)
Age Group (Years)	
<60	157 (95.2)
≥60	8 (4.8)
Body Mass Index	
Severely Underweight (≤16.49)	10 (6.1)
Underweight (16.5–18.49)	9 (5.5)
Normal (18.5–22.9)	58 (35.2)
Overweight (23–24.9)	18 (10.9)
Obese (≥25)	54 (32.7)
No data	16 (9.7)
Autoimmune Diagnosis	
SLE	90 (54.5)
Sjogren’s syndrome	55 (33.5)
Antiphospholipid syndrome (APS)	38 (23)
Autoimmune vasculitis	9 (5.5)
Autoimmune thyroid diseases	6 (3.6)
Rheumatoid arthritis	5 (3.03)
Inflammatory Bowel Disease	5 (3.03)
Scleroderma/Systemic sclerosis	4 (2.4)
Autoimmune Haemolytic Anemia (AIHA)	3 (1.82)
Mixed Connective Tissue Disease (MCTD)	3 (1.82)
Myasthenia gravis	3 (1.82)
Other autoimmune diseases *	43 (26.06)
Routine Immunosuppressant therapy	
Steroid	118 (71.5)
≥10 mg of Prednisone-equivalent per day	43 (36.4)
<10 mg of Prednisone-equivalent per day	72 (61.0)
No data	3 (2.5)
Hydroxychloroquine	68 (41.2)
Mycophenolate mofetil/mycophenolic acid (MMF/MPA)	68 (41.2)
Azathioprine	11 (6.7)
Cyclophosphamide	2 (1.2)
Cyclosporine	2 (1.2)
Tacrolimus	3 (1.8)
Methotrexate	3 (1.8)
Rituximab	1 (0.6)
Routine medications	
ACE-Inhibitor	9 (5.5)
Angiotensin Receptor Blocker (ARB)	12 (7.3)
Antiplatelet	22 (13.3)
Anticoagulant	14 (8.5)
Vitamin D3 supplement	45 (27.3)
COVID-19 Vaccination Status	
Vaccinated	70 (42.4)
CoronaVac	28 (40)
BNT162b2 (Pfizer-BioNTech)	17 (24.3)
mRNA-1273 (Moderna)	8 (11.4)
ChAdOx1 nCov-19 (Oxford-AstraZeneca)	2 (2.9)
Not vaccinated	95 (57.6)
Time of vaccination	
Before COVID-19 infection	23 (32.8)
After COVID-19 infection	34 (48.5)
No data	13 (18.6)
Comorbidities	
Hypertension	30 (18.3)
Diabetes Mellitus	9 (5.5)
Chronic Kidney Disease (CKD)	18 (11)
Cardiovascular Disease (CVD)	13 (7.9)
COVID-19 Severity	
Moderate-Severe	34 (20.6)
Mild	131 (79.4)
COVID-19 Hospitalization	
Hospitalization in isolation ward or ICU	61 (36.9)
Self-isolation	104 (63)
COVID-19 Outcome	
Alive	161 (97.6)
Deceased	4 (2.4)

* Other autoimmune diseases: undifferentiated connective tissue disease, neuromyelitis optica, transverse myelitis, psoriasis.

**Table 2:** Factors associated with severity, hospitalization, and mortality among patients with autoimmune diseases (*n* = 165).

Patient Variables	Severity						Hospitalization						Outcome			
	Moderate-Severe N = 34	Mild N = 131	Bivariate		Multivariate		Isolation Ward/ICU N = 61	Self-Isolation N = 104	Bivariate		Multivariate		Deceased N = 4	Alive N = 161	Bivariate	
	*n* (%)	*n* (%)	OR (95% CI)	*p*	OR (95% CI)	*p*	*n* (%)	*n* (%)	OR (95% CI)	*p*	OR (95% CI)	*p*	*n* (%)	*n* (%)	OR (95% CI)	*p*
Sex																
Female	33 (21.4)	121 (78.6)	2.73	0.295			57 (37.0)	97 (63.0)	1.028	0.618			3 (1.9)	151 (98.1)	0.19	0.243
Male	1 (9.1)	10 (90.9	(0.34–22.08)				4 (36.4)	7 (63.6)	(0.29–3.67)				1 (9.1)	10 (90.9)	(0.02–2.09)	
Age group																
<60	32 (20.4)	125 (79.6)	0.77	0.518			58 (36.9)	99 (63.1)	0.97	0.622			3 (1.9)	154 (98.1)	0.14	0.182
≥60	2 (25.0)	6 (75.0)	(0.15–3.99)				3 (37.5)	5 (62.5)	(0.23–4.24)				1 (12.5)	7 (87.5)	(0.01–1.48)	
Autoimmune Diagnosis																
SLE																
(+)	19 (21.1)	71 (78.9)	1.07	1.000			37 (41.1)	53 (58.9)	1.48	0.227	1.13	0.836	4 (4.4)	86 (95.6)	-	0.086
(−)	15 (20.0)	60 (80.0)	(0.50–2.29)				24 (32.0)	51 (68.0)	(0.78–2.82)		(0.36–3.51)		0 (0)	75 (100)		
Sjogren’s syndrome																
(+)	10 (18.2)	45 (81.8)	0.79	0.734			17 (30.9)	38 (69.1)	0.67	0.254			0 (0)	55(100)	-	0.194
(−)	24 (21.8)	86 (78.2)	(0.35–1.81)				44 (40.0)	66 (60.0)	(0.34–1.34)				4 (3.6)	106 (96.4)		
APS																
(+)	10 (26.3)	28 (73.7)	1.53	0.445			18 (47.4)	20 (52.6)	1.76	0.130	2.36	0.100	1 (2.6)	37 (97.4)	1.11	0.653
(−)	24 (18.9)	103 (81.1)	(0.66–3.58)				43 (33.9)	84 (66.1)	(0.84–3.67)		(0.85–6.54)		3 (2.4)	124 (97.6)	(0.11–11.06)	
Autoimmune thyroid																
(+)	1 (16.7)	5 (83.3)	0.76	0.640			1 (16.7)	5 (83.3)	0.33	0.279			0 (0)	6 (100)	-	0.861
(−)	33 (20.8)	126 (79.2)	(0.86–6.76)				60 (37.7)	99 (62.3)	(0.04–2.89)				4 (2.5)	155 (97.5)		
Autoimmune vasculitis																
(+)	0 (0)	9 (100)	-	0.118			4 (44.4)	5 (55.9)	1.39	0.440			0 (0)	9 (100)	-	0.797
(−)	34 (21.8)	122 (78.2)					57 (36.5)	99 (63.5)	(0.36–5.38)				4 (2.6)	152 (97.4)		
COVID-19 Vaccination Status																
Not vaccinated	26 (27.4)	69 (72.6)	2.92	0.021 *	5.65	0.019 *	42 (44.2)	53 (55.8)	2.13	0.025 *	2.63	0.050	4 (4.2)	91 (95.8)	-	0.107
Vaccinated	8 (11.4)	62 (88.6)	(1.23–6.92)		(1.32–24.16)		19 (27.1)	51 (72.9)	(1.09–4.13)		(0.99–6.95)		0 (0)	70 (100)		
Comorbidities																
Hypertension																
(+)	8 (24.2)	25 (75.8)	1.305	0.736			14 (42.4)	19 (57.6)	1.33	0.468			1 (3)	32 (97)	1.34	0.594
(−)	26 (19.7)	106 (80.3)	(0.53–3.22)				47 (35.6)	85 (64.4)	(0.61–2.89)				3 (2.3)	129 (97.7)	(0.13–13.35)	
Diabetes mellitus																
(+)	5 (55.6)	4 (44.4)	5.47	0.019 *	4.23	0.102	6 (66.7)	3 (33.3)	3.67	0.064	3.39	0.197	1 (11.1)	8 (88.9)	6.37	0.203
(−)	29 (18.6)	127 (81.4)	(1.38–21.65)		(0.75–23.85)		55 (35.3)	101 (64.7)	(0.88–15.26)		(0.53–21.78)		3 (1.9)	153 (98.1)	(0.59–68.34)	
CKD																
(+)	3 (16.7)	15 (83.3)	0.75	0.468			10 (55.6)	8 (44.4)	2.35	0.084	3.06	0.061	2 (11.1)	16 (88.9)	8.17	0.059
(−)	31 (21.1)	116 (78.9)	(0.20–2.75)				51 (34.7)	96 (65.3)	(0.87–6.31)		(0.95–9.88)		2 (1.4)	145 (98.6)	(1.22–54.48)	
CVD																
(+)	6 (40.0)	9 (60.0)	2.90	0.060	7.22	0.022 *	7 (46.7)	8 (53.3)	1.56	0.415			2 (13.3)	13 (86.7)	11.38	0.042 *
(−)	28 (18.7)	122 (81.3)	(0.95–8.83)		(1.34–39.00)		54 (36.0)	96 (64.0)	(0.53–4.52)				2 (1.3)	148 (98.7)	(1.48–87.58)	
Obesity																
(+)	11 (20.4)	43 (79.6)	1.05	1.000			16 (29.6)	38 (70.4)	0.65	0.241	0.54	0.226	0 (0)	54 (100)	-	0.262
(−)	19 (19.6)	78 (80.4)	(0.46–2.41)				38 (39.2)	59 (60.8)	(0.32–1.33)		(0.19–1.46)		3 (3.1)	94 (96.9)		
Routine Immunosuppressant/Immunomodulator Therapy																
Corticosteroid																
(+)	23 (19.5)	95 (80.5)	0.79	0.728			44 (37.3)	74 (63.2)	1.05	1.000			4 (3.4)	114 (96.6)	-	0.668
(−)	11 (23.4)	36 (76.6)	(0.35–1.79)				17 (36.2)	30 (62.5)	(0.52–2.12)				0 (0.0)	47 (100)		
Corticosteroid dosage, prednisone equivalent																
≥10 mg/day	13 (30.2)	30 (69.8)	3.47	0.010 *	3.01	0.037 *	26 (60.5)	17 (39.5)	5.35	<0.001 *	5.36	<0.001 *	2 (4.7)	41 (95.3)	-	0.140
<10 mg/day	8 (11.1)	64 (88.9)	(1.29–9.25)		(1.06–8.48)		16 (22.2)	56 (77.8)	(2.34–12.23)		(2.10–13.65)		0 (0)	71 (100)		
Azathioprine																
(+)	0 (0)	11 (100)	-	0.072			4 (36.4)	7 (63.6)	0.97	0.618			0 (0)	11 (100)	-	0.757
(−)	34 (22.1)	120 (77.9)					57 (37.0)	97 (63.0)	(0.27–3.47)				4 (2.6)	150 (97.4)		
Hydroxychloroquine																
(+)	11 (16.2)	57 (83.8)	0.62	0.239	2.53	0.117	18 (26.5)	50 (73.5)	0.45	0.011 *	1.69	0.315	1 (1.5)	67 (98.5)	0.47	0.453
(−)	23 (23.7)	74 (76.3)	(0.28–1.38)		(0.79–8.09)		43 (44.3)	54 (55.7)	(0.23–0.88)		(0.60–4.77)		3 (3.1)	94 (96.9)	(0.05–4.59)	
MMF/MPA																
(+)	13 (19.1)	55 (80.9)	0.85	0.692			21 (30.9)	47 (69.1)	0.64	0.175			2 (2.9)	66 (97.1)	1.44	0.547
(−)	21 (21.6)	76 (78.4)	(0.39–1.85)				41 (41.2)	57 (58.8)	(0.33–1.22)				3 (2.1)	95 (97.9)	(0.19–10.47)	
Cyclophosphamide																
(+)	1 (50)	1 (50)	3.94	0.371			1 (50.0)	1 (50.0)	1.71	0.604			0 (0)	2 (100)	-	0.952
(−)	33 (20.2)	130 (79.8)	(0.24–64.65)				60 (36.8)	103 (63.2)	(0.10–27.95)				4 (2.5)	159 (97.5)		
Cyclosporine																
(+)	0 (0)	2 (100)	-	0.629			0 (0.0)	2 (100)	-	0.396			0 (0)	2 (100)	-	0.952
(−)	34 (20.9)	129 (79.1)					61 (37.4)	102 (62.6)					4 (2.5)	159 (97.5)		
Tacrolimus																
(+)	1 (33.3)	2 (66.7)	1.95	0.502			3 (100)	0 (0)	-	0.049 *			1 (33.3)	2 (66.7)	26.5	0.071
(−)	33 (20.4)	129 (79.6)	(0.17–22.22)				58 (35.8)	104 (64.2)					3 (1.9)	159 (98.1)	(1.86–378.23)	
Methotrexate																
(+)	0 (0)	3 (100)	-	0.489			0 (0.0)	3 (100)	-	0.248			0 (0)	3 (100)	-	0.929
(−)	34 (21.0)	128 (79)					61 (37.7)	101 (62.3)					4 (2.5)	158 (97.5)		
Routine Medications																
ACE-Inhibitor																
(+)	1 (11.1)	8 (88.9)	0.47	0.412			4 (44.4)	5 (55.6)	1.39	0.440			0 (0)	9 (100)	-	0.797
(−)	33 (21.2)	123 (78.8)	(0.05–3.86)				57 (36.5)	99 (63.5)	(0.36–5.38)				4 (2.6)	152 (97.4)		
ARB																
(+)	3 (25.0)	9 (75)	1.31	0.466			4 (33.3)	8 (66.7)	0.84	0.525			1 (8.3)	11 (91.7)	4.54	0.263
(−)	31 (20.3)	122 (79.7)	(0.33–5.14)				57 (37.3)	96 (62.7)	(0.24–2.92)				3 (2.0)	150 (98.0)	(0.44–47.40)	
Antiplatelet																
(+)	4 (17.4)	19 (82.6)	0.78	0.463			9 (39.1)	14 (60.9)	1.11	0.817			0 (0)	23 (100)	-	0.545
(−)	30 (21.1)	112 (78.9)	(0.25–2.48)				52 (36.6)	90 (63.4)	(0.45–2.75)				4 (2.8)	138 (97.2)		
Anticoagulant																
(+)	3 (21.4)	11 (78.6)	1.05	0.582			5 (35.7)	9 (64.3)	0.94	0.919			1 (7.1)	13 (92.9)	3.79	0.301
(−)	31 (20.5)	120 (79.5)	(0.27–4.02)				56 (37.1)	95 (62.9)	(0.30–2.95)				3 (2.0)	148 (98.0)	(0.37–39.12)	
Vitamin D3 supplement																
(+)	7 (15.6)	38 (84.4)	0.63	0.444			11 (24.4)	34 (75.6)	0.45	0.041 *	2.63	0.129	0 (0)	45 (100)	-	0.276
(−)	27 (22.5)	93 (77.5)	(0.25–1.58)				50 (41.7)	70 (58.3)	(0.21–0.98)		(0.75–9.18)		4 (3.3)	116 (96.7)		

ICU: Intensive Care Unit; SLE: Systemic lupus erythematosus; APS: Antiphospholipid syndrome; CKD: chronic kidney disease; CVD: cardiovascular disease; MMF/MPA: Mycophenolate mofetil/mycophenolic acid; ARB: Angiotensin-Receptor Blocker. * *p* < 0.05.

**Table 3:** Characteristics of the COVID-19 deaths in patients with autoimmune diseases.

Variables	Case 1	Case 2	Case 3	Case 4
Age (in years)	62	27	22	33
Sex	Female	Female	Female	Male
Body mass index (category)	20.44 (underweight)	No data	13.67 (severely underweight)	23.88 (normal)
Autoimmune Diagnosis	SLE	SLE, APS	SLE	SLE
Comorbidities	Cardiovascular disease, chronic kidney disease	Dialysis-dependent chronic kidney disease, hypercoagulable state, deep vein thrombosis	Cardiovascular disease, chronic kidney disease	Hypertension, Diabetes Mellitus, Tuberculosis
COVID-19 Vaccination	Not vaccinated	Not vaccinated	Not vaccinated	Not vaccinated
First COVID-19 Diagnosis	June 2020	September 2020	June 2021	February 2022
Second COVID-19 Diagnosis	-	-	February 2022	-
COVID-19 Severity	Severe	Severe	Severe	Moderate
Immunosuppressant Therapy before COVID-19 diagnosis	Corticosteroid 5 mg prednisone-equivalent per day; MPA	Not visiting the doctor nor taking autoimmune treatment since the start of pandemic	Corticosteroid 40 mg prednisone-equivalent per day; MPA; Tacrolimus	Corticosteroid 60 mg prednisone-equivalent per day; Rituximab; MPA
Immunosuppressant therapy during COVID-19 infection	MPA discontinued	-	MPA and tacrolimus discontinued	MPA discontinued
Hospitalization	Isolation room	ICU	Isolation Room	Isolation Room
Time to Death (days)	7	12	1	13
Cause of Death	Cardiogenic shock	Respiratory failure	Septic shock	Hypovolemic shock due to active haemoptysis

SLE: Systemic Lupus Erythematosus, APS: Antiphospholipid syndrome MPA: Mycophenolic acid, ICU: Intensive Care Unit.

## Data Availability

The data used are available from the corresponding author upon request.
